# Human Leukocyte Antigen Diversity: A Southern African Perspective

**DOI:** 10.1155/2015/746151

**Published:** 2015-08-12

**Authors:** Mqondisi Tshabalala, Juanita Mellet, Michael S. Pepper

**Affiliations:** Department of Immunology and Institute for Cellular and Molecular Medicine, Faculty of Health Sciences, University of Pretoria, P.O. Box 2034, Pretoria 0001, South Africa

## Abstract

Despite the increasingly well-documented evidence of high genetic, ethnic, and linguistic diversity amongst African populations, there is limited data on human leukocyte antigen (HLA) diversity in these populations. HLA is part of the host defense mechanism mediated through antigen presentation to effector cells of the immune system. With the high disease burden in southern Africa, HLA diversity data is increasingly important in the design of population-specific vaccines and the improvement of transplantation therapeutic interventions. This review highlights the paucity of HLA diversity data amongst southern African populations and defines a need for information of this kind. This information will support disease association studies, provide guidance in vaccine design, and improve transplantation outcomes.

## 1. Introduction

The human leukocyte antigen (HLA) complex on chromosome 6, also known as the major histocompatibility complex (MHC) in all mammals, consists of highly polymorphic genes whose protein products present antigens to T cells as part of an immune response to infections [1, 2]. HLA molecules also impact on the development and effectiveness of vaccines and play a determining role in the outcomes of transplantation [3–10].

The World Health Organization (WHO) indicates that there is a high burden of disease in southern Africa, especially communicable diseases such as HIV/AIDS, TB, and malaria [11]. Despite the increasingly well-documented high genetic diversity observed amongst human populations in southern Africa [12], there is limited information on HLA diversity [8]. Understanding HLA diversity in these populations will provide insight into HLA disease associations and may help in vaccine development. Transplantation as a therapeutic intervention requires strict HLA allele matching between donors and recipients to reduce rejection and the incidence of graft versus host disease (GVHD). Good clinical outcomes in transplant recipients are observed in cases of high resolution HLA matching [13, 14], with the number of mismatches correlating with the risk of rejection and/or GVHD [15–17]. It is currently very difficult to match donor-recipient pairs in bone marrow registries in southern Africa, partly because of the great genetic diversity in this population. A recent study identified Black and Caucasian South African population-specific alleles [18], highlighting the need to investigate HLA diversity amongst southern Africans to improve global representation in the International ImMunoGeneTics information system (IMGT)/HLA database [1, 2]. HLA typing methods use the IMGT/HLA database as a reference; it is thus difficult to match individuals who have alleles which are not captured in the database.

HLA typing methods have evolved from low resolution serology typing to high resolution DNA sequencing based technologies (SBT). Despite high resolution, SBT has limitations of mostly typing certain exons within the HLA loci [19]. The antigen-binding grooves encoded by exons 2 and 3 (class I) and exon 2 (class II) are routinely sequenced in most laboratories, thereby giving partial sequences of about 10% of the reported alleles [19]. Another potential source of ambiguity in SBT HLA typing is the* cis/trans* assignment of DNA bases in a heterogeneous sample [20], yielding limited resolution data and thereby making it difficult to assign HLA allele types. It is possible to sequence the entire HLA region with current methods, but at a very high cost and a need for expert analysis. There have been advances in the use of next generation sequencing (NGS) in HLA typing to improve coverage of the HLA gene loci by high throughput, while at the same time reducing ambiguity associated with SBT typing [19, 21, 22]. To fully appreciate the NGS HLA typing tool, there is need for a complete HLA allele database [21] highlighting the need to quantify HLA diversity in the genetically diverse southern African populations [23].

African populations have been shown to be genetically diverse [12] and are believed to be the cradle of humankind [24, 25]. In general, genetic diversity of African populations is poorly understood [8] thereby limiting our understanding of human health and susceptibility to diseases, hence the need for further analysis/evaluation to map disease association and theraupetic gene targets. Despite the general similarities in culture and shared geographical location, genetic differences exist among populations at every 1000 base pairs [26, 27]. In this review, we examine available HLA diversity data in southern Africa with a view to understanding disease burden, planning registry recruitment and donor-recipient matching, and providing insights into the evolution of the ethnic and linguistic diversity in this region. This review specifically focuses on classical HLA diversity in southern African countries (characterized by genetically, culturally, and linguistically diverse Bantu ethnicities and admixed populations [28–31]) herein defined as Zambia, Malawi, Zimbabwe, Mozambique, Angola, Namibia, Botswana, South Africa, Lesotho, and Swaziland.

## 2. HLA Diversity 

There are an ever increasing number of HLA alleles, reflecting the rate of discovery of the diversity of the gene loci [1, 2]. There are currently 13412 HLA alleles described by the HLA nomenclature and included in the IMGT/HLA database (based on IMTG/HLA 3.21.0 release, 06 July 2015), with HLA-B having the highest number of alleles (3977) [32]. HLA genetic variation does not vary in an individual's lifetime, but high diversity is observed at the population level [1, 2, 33–38]. High HLA allelic diversity in humans is reflected by the high number of pseudogenes and can be explained by natural selection and coevolution with pathogens. There is an advantage of HLA diversity related to pathogen-derived peptide presentation to effector T cells: heterozygous individuals can potentially present more antigens than homozygotes for the different HLA alleles (heterozygosity advantage) [33, 39]. In nonhuman species, low MHC diversity has been observed in several species (Tasmanian devils, cheetah, and panda) and has been associated with disease susceptibility in some Tasmania devils [40], highlighting the advantage of HLA diversity in presenting many different antigens to effector cells of the immune system.

Prugnolle et al. suggested that up to 39% of observed HLA class I diversity was due to geographical distance (and consequently human migration history) from the source of modern humans (assumed to be Ethiopia in this study), with the unaccounted source of diversity most likely being from pathogen driven selection [41]. Generally, populations exposed to a high pathogen burden show high HLA diversity, and there is a decreasing HLA diversity away from Africa (geographically measured by landmasses away from Africa) [41]. In related studies, microsatellite data has suggested that geographic distance from East Africa (probable source of modern humans) explains about 85% of a decreasing genetic diversity within human populations from the source (reviewed in [42]). Interestingly, HLA-C is less expressed on cell surfaces; hence its diversity is least likely to be driven by viral pathogens (reviewed in [41]). It is historically accepted that TB was a major selective pressure in the evolution of Western European populations [43], with malaria acting on African populations [44]. These pathogens exerted a high selective pressure mostly on genes of the immune system (particularly those involved in protective immunity).

There is growing evidence for positive selection being responsible for maintaining HLA polymorphisms, most likely due to overdominant selection (heterozygote advantage) which maintains allelic lineages for much longer periods of time than neutral polymorphisms [38, 45–47]. Globally, HLA diversity seems to be highest within populations than between populations (evidenced by major differences amongst continents) [1, 2, 35, 48]. Several studies have highlighted alternative splicing of HLA class I genes giving rise to diverse isoforms [49] which might contribute to this diversity. For example, alternative splicing to exclude exon 5 has been reported to give rise to several isoforms of HLA-A and -B [50]. Alternative splicing in other HLA class I exons has also been reported [51] including the nonclassical HLA-G gene [52].

Other mechanisms of HLA diversity generation include point mutations (substitution, deletion, and insertion): gene conversion (unidirectional gene transfer) and gene cross over (bidirectional gene transfer). Gene cross over, which is a form of recombination that can be intra/inter-HLA loci during meiosis, enables exchange of genetic material linked to the generation of novel alleles in offspring as described by Carrington [53]. Other recombination events include gene conversion, a bidirectional donation of DNA between two homologous chromosomes. A recent study reports novel HLA alleles resulting from (a) nonsynonymous amino acid change (HLA B^*∗*^41:21, HLA DQB1^*∗*^02:10, HLA QA1^*∗*^01:12); (b) deletion leading to frame shift (HLA A^*∗*^01:123N); (c) intralocus gene conversion (HLA B^*∗*^35:231, HLA B^*∗*^53:31); and (d) interlocus gene conversion (HLA C^*∗*^07:294) [54]. It is important to note the low frequency of interlocus generated alleles as reported by several other studies as reviewed by Adamek et al. [54].

### 2.1. HLA Diversity in Transplantation and Transfusion

The human immune system uses HLA's uniqueness in every individual to recognize self from nonself; hence the body only mounts an immune response against foreign cells/molecules under normal conditions. Transplantation as a therapeutic intervention matches donor and recipient HLA molecules to decrease the likelihood of rejection [33]. The likelihood of two individuals having identical HLA molecules on all loci is very low, except for siblings, who have a 25% chance of being HLA-identical as a result of HLA molecules being codominantly expressed and inherited as haplotypes from both parents. The degree of HLA matching is a predictor of clinical outcome.

GVHD is an immunocompetent donor T cell mediated response against the recipient's immune system which is common in unmatched donor-recipient pairs. Acute GVHD can be reduced by donor T cell depletion, but this increases the risk of rejection, malignant disease relapse, and impaired immune recovery [63, 64]. In addition to HLA matching, killer-cell immunoglobulin-like receptors (KIRs) have been documented to affect the clinical outcome of allogeneic transplantation [65–68]. In severe immunocompromised individuals, allogeneic transfusion with immune competent T cell-containing blood products might lead to transfusion associated GVHD. Transfusion related lung injury (TRALI) is an anti-HLA (mostly class I [69, 70]) antibody related complication which may be fatal. Anti-HLA class II antibodies induce TRALI through monocyte and subsequent neutrophil activation [69, 71]. Anti-HLA class I antibodies have been reported to be a cause of neonatal alloimmune thrombocytopenia together with platelet-derived specific antigens [72]. It is critical to know the population HLA diversity in order to improve donor-recipient matching in both transplantation and transfusion therapeutic interventions. Diversity data informs decision making in transplantation and transfusion aimed at reducing rejection while at the same time improving the outcome of the intended therapeutic intervention. Recruitment of donors from minority or underrepresented populations might help to improve HLA diversity in registries [34] which improves the chances of donor-recipient matching.

### 2.2. HLA Diversity in Human Disease Associations

The high disease burden in southern Africa [11] offers a unique opportunity to study HLA disease association [8]. Several autoimmune conditions have been directly associated with specific class I and II HLA alleles, including rheumatoid arthritis, multiple sclerosis, ankylosing spondylitis, and Grave's disease, as reviewed by Trowsdale and Knight [73]. Several alleles have been associated with varying rates of HIV disease progression [4, 39, 74–76], susceptibility, transmission, and treatment outcomes (reviewed in [76]). HLA has likewise been associated with malaria [6] and TB susceptibility and protection [77] in various populations. In another example, although not directly related to southern Africa, the HLA-B locus has been linked to fatal and nonfatal Sudanese Ebola strains. Thus, HLA-B^*∗*^67 and -B^*∗*^15 have been associated with fatal outcomes and B^*∗*^07 and B^*∗*^14 have been associated with nonfatal Ebola infections [78].

Haplotype analysis gives information on disease/condition associated alleles, which are assumed to be inherited as blocks due to strong linkage disequilibrium [79]. HLA alleles can be imputed from analyzing identity by descent (IBD) patterns within the HLA region of specific populations. This approach leverages on the observation that chromosomes with high IBD within MHC most likely share the same alleles. Haplotype analysis or SNP-based HLA allele imputation is important for disease association studies but will not replace classical HLA typing for transplantation applications where a high degree of haplomatching is required for a good clinical outcome [80]. Currently several imputation methods are available to type HLA genes in silico and to fine-map associations within classical HLA genes [80]. Unfortunately, limited HLA diversity data from populations such as those in southern Africa make this difficult [80].

### 2.3. HLA Diversity in Population Studies

There is documented evidence of geographical distribution of human genetic variation, which helps to understand human evolution, migration and adaptation to different environments and pathogens [81]. Several efforts aimed at understanding global human genetic diversity including the Hap Map Project [82], 1000 Genomes Project [83], and recently the African Genome Variation Project [31]; however, all of these have limited information on southern African populations. Some African genetic diversity studies have focused on targeted populations like hunter gatherers [84, 85] or have had very limited sample size [86] and are therefore not representative of southern Africa. The low representation of southern African genetic data in global efforts makes it difficult to use the currently available reference panels for these populations, especially in disease association studies [31]. This suggests that targeted HLA sequencing of these diverse populations is necessary to improve their representation in reference panels.

There are marked differences in HLA diversity distribution globally, with geographically separated regions showing varying degrees of diversity [35, 41, 42, 48]. Most HLA loci show high allele numbers across populations [35, 87]. HLA DPA1 has the least number of alleles (40 as of July 2015) [88] compared to other classical HLA loci (e.g., HLA DQB1 which has 807 alleles). This is generally due to the fact that DPB1 loci are not routinely sequenced for transplantation purposes as are other HLA genes. The global distribution of HLA diversity provides insight into human migration patterns and could help understand past pathogen exposures [38]. As an example, HLA studies have been used to trace the spread of modern humans from East Africa and model for coevolution of genes and languages in Africa [89]. Interpretation of HLA in population studies can be improved by extensive knowledge of HLA diversity in these populations.

### 2.4. Contemporary Studies on HLA Diversity in Southern Africa

To highlight the paucity of HLA diversity data in southern Africa, this review used a comprehensive literature search for previously published work on HLA diversity together with the Allele Frequency Net Database (AFND) to determine the information in the public domain. The key search terms for articles were “HLA AND genetic diversity AND southern Africa”. Allele frequency data from AFND was extracted for sub-Saharan African countries, from which southern African data was compiled (Supplementary Table S1,in Supplementary Material available online at http://dx.doi.org/10.1155/2015/746151).  Table 1 summarizes allele frequency data from the AFND web search (http://www.allelefrequencies.net/) [48] used in this review. The AFND is a public global database of alleles, genotypes, and haplotype frequencies of HLA and KIRs from different studies, reports, and proceedings of international workshops in immunogenetics and histocompatibility. HLA data is generated by different typing methods but is curated in the database in accordance with the updated IMGT/HLA guidelines (this review used the 3.15.0 release, 17 January 2014) [1, 2, 35, 48]. For this review, only positive allele frequencies from all ethnic groups within sub-Saharan Africa were extracted from the database (http://www.allelefrequencies.net/) [35, 48]. The number of alleles reported in Mozambicans, Black South Africans, Caucasian South Africans, Tamil South Africans, Zulu South Africans, Tswana South Africans, Zambians, and Shona Zimbabweans, respectively, was 18, 33, 25, 16, 37, 15, 20, and 32 alleles for HLA-A and 25, 30, 41, 23, 45, 14, 29, and 46 alleles for HLA-B. HLA-C alleles were only reported for Black South Africans (28 alleles), Caucasian South Africans (29 alleles), Tamil South Africans (21 alleles), Zambians (12 alleles), and Shona Zimbabweans (24 alleles). All HLA class II alleles in the AFND were only reported for Shona Zimbabweans and South African Vendas as summarized in Supplementary Table S1. Tables 1 and 2 summarize the selected allele frequencies from southern African populations and the total number of classical HLA alleles reported across different global regions as defined in the AFND [35, 48], respectively.

South Africa had the highest number of HLA data sets from the AFND compared to other southern African countries (Table 1(a)). Some southern African countries (Angola, Lesotho, Malawi, Namibia, and Swaziland) have no HLA data available (Table 1(a)). As summarized in Tables 1(b) and 1(c), HLA-A^*∗*^30 and its derivatives (A^*∗*^30:01, A^*∗*^30:02) are common in black populations (Mozambicans, Black South Africans, Zulus, Tswanas, Zambians, and Zimbabwean Shonas). Caucasians and Tamils had a completely different HLA-A allele frequency distribution compared to the other populations. HLA-A^*∗*^02:01:01 was most frequent (0.26) in South African Caucasians, as has been reported by Solberg et al. (HLA-A^*∗*^02:01) in European (27%) and white American (20%) populations [90]. This suggests that South African Caucasians have a common ancestry with the Europeans and Americans, with the A^*∗*^02:01 allele and its derivatives being restricted mostly to white populations. For the HLA-B locus, B^*∗*^58 (B^*∗*^58:02, B^*∗*^58:01) was most common in Mozambicans, Black South Africans (including Zulus and Tswanas) as highlighted in Tables 1(b) and 1(c). All HLA-B allele frequencies were less than 0.1 in Black South Africans and Shonas. All HLA-C frequencies were less than 0.2, with C^*∗*^06:02 being commonly high in Black South Africans and Tamils. Although more than ten years old (2004), the study by Cao et al. identified A^*∗*^02:02, A^*∗*^34:02, A^*∗*^36:01, A^*∗*^74:01, B^*∗*^15:03, B^*∗*^42:01, B^*∗*^53:01, B^*∗*^57:03, and B^*∗*^58:02 as unique African alleles. Recently, diverse and novel HLA alleles have been reported in sub-Saharan populations, for example, HLA class II as reviewed in Ayele et al. [91] and HLA class I as described by Paximadis et al. [18], to further support high genetic diversity in Africans and intra-African diversity. Interestingly five new class I alleles (A^*∗*^30:01:02, A^*∗*^30:02:02, A^*∗*^68:27, B^*∗*^42:06, and B^*∗*^45:07) were reported in a recent South African study [18]. Additionally, Shepherd et al. recently reported an overrepresentation of HLA-A^*∗*^02:01, -A^*∗*^34:02, and -B^*∗*^58:02 in HIV negative controls in Zimbabwe [92] compared to the HIV positive group, which supports the earlier notion of African specific alleles.

The AFND reports very few HLA class II alleles amongst southern African populations; only Zimbabwean Shonas and Black South Africans [18] had HLA-DP data. The reported allele frequencies (Tables 1(b) and 1(c)) for the DP locus were most frequent DPB1^*∗*^01:01:01 (0.355) in Shona Zimbabweans and DPB1^*∗*^13:01 (0.148) in Black South Africans; and least frequent DPB1^*∗*^01:01:02, DPB1^*∗*^02:02, DPB1^*∗*^62:01, DPB1^*∗*^65:01, and DPB1^*∗*^80:01 (0.002) in Shona Zimbabweans. No alleles were reported for the DPA1 and DQA1 loci. The DQB1 locus was reported only in Botswana, Black South Africans, Shona Zimbabweans, and Venda South Africans. DQB1^*∗*^06 in Black South Africans was the most frequent (0.555) with DQB1^*∗*^06:15 in Shona Zimbabweans being least frequent (0.002). DRB1 alleles were reported in all the studied populations except in some South Africans (Tswana, Tamil, and Zulu). The most frequent allele was DRB1^*∗*^11 (0.366) in Black South Africans, while the least frequent ones were DRB1^*∗*^16 (0.002) in Mozambicans and DRB1^*∗*^03, DRB1^*∗*^04:04, DRB1^*∗*^12:04, DRB1^*∗*^13, and DRB1^*∗*^15:01 (all at 0.002) in Shona Zimbabweans.

The number of classical HLA alleles (Table 2) varies greatly in each geographical region, with North Africa having the highest number of AFND reported alleles globally, and sub-Saharan Africa (including southern Africa) in the top 5. In terms of HLA class II alleles, sub-Saharan Africa falls in the bottom 5 regions (with the least number of alleles, Table 2) for most of the HLA loci (DQA1, DQB1, and DRB1). The DP locus generally has fewer numbers of reported alleles globally (http://www.allelefrequencies.net/) [35, 48]. Interestingly, more than 50% of HLA class I alleles reported for sub-Saharan Africa are in southern Africa (Table 2), further highlighting diversity in this region. No HLA-DPA1 alleles were reported by the AFND in southern Africa, with less than 50% of the other class II alleles reported in sub-Saharan Africa coming from southern Africa.

The number of southern African HLA studies in the AFND is relatively low, reflecting the underrepresentation of this region. The data currently available is mostly low resolution with low sample numbers and is not a true reflection of HLA diversity in the southern African context. This highlights the need for continual submission of southern African HLA diversity data to centralized databases like the AFND. The few studies from southern Africa also highlight the knowledge gap on HLA diversity in this region in this era of high resolution typing. Several HLA disease association studies with allele frequency data have been reported in the region [7, 59, 93–95]; these frequencies might not be a true reflection of the general population owing to the confounding effect of the diseases. Allele frequency is highly dependent on sample size and hence might not give a clear picture of HLA diversity.

## 3. Concluding Remarks

There is limited data on HLA diversity in southern Africa, with most having been generated from disease association studies and which is therefore not a true reflection of the general population. It is often difficult to assign causality of a specific HLA allele to an infection/condition, because of linkage disequilibrium and other factors such as selection pressure, which are dependent on the condition/infection and the other arms of the immune system which are HLA independent [96]. As evidenced by the HIV example, several HLA-B alleles have been associated with control of viremia [4, 97, 98] yet some individuals with these protective alleles develop AIDS (fail to control the virus) [99]. Recently Chen et al. showed that HLA B^*∗*^27 restricted CD8 T cells had variable viral replication inhibition capabilities in HIV controllers* versus* progressors due to a modulation by specific T cell receptor clonotypes [5]. There are few high resolution HLA datasets from southern African populations [1, 2, 35, 48] despite growing advancement in NGS HLA typing.

HLA diversity data forms the cornerstone of population-specific vaccine development, and taking into consideration the high disease burden in southern Africa, information of this nature is particularly important in this region [11]. This review highlights the paucity of information on HLA genotypic data and documents the extent of HLA diversity data from the southern African perspective based on the limited data available. This underpins an urgent need for HLA data from the general populations in this region and for studies which elucidate the extent of this diversity. There is a need to build an HLA diversity resource for southern Africa (or Africa as a whole) such as, for example, the HLA-net (a European network) [100] which focuses on HLA diversity and its applications in histocompatibility, transplantation, epidemiology, and population genetics. This network has developed analysis pipelines and guidelines for HLA diversity data for mostly European populations [100, 101]. It is thus possible to build such a resource for the genetically diverse and disease burdened African continent to be used as a guideline for future studies including donor recruitment strategies [34], population studies [38, 89, 101], and disease association studies [6, 8, 77, 78].

Furthermore, advancement in HLA typing methods such as NGS will help to finely investigate HLA diversity, as previous strategies have targeted a few exons per locus thereby missing some of medically important variants outside the typed regions.

An understanding of HLA diversity will provide insight into allele frequency dependent selection fitness which varies between populations. This might help understand the high disease burden (especially with regard to HIV) and form the basis of vaccine development for the many infectious diseases as well as in the planning of vaccine clinical trials in the region. The paucity of HLA data from this region is a major hurdle in vaccine design [7]. Brumme et al. highlight, for example, the need to elucidate HLA-restricted CTL responses in HIV vaccine design [102]. HLA class II antigens presented to CD4^+^ T cells induce B cells leading to an antigen specific humoral immune response [103]. HLA class II alleles have been associated with humoral immune response inducing vaccines for malaria [104], active anticancer immunotherapy [105], and HIV [106]. The combined use of HLA class II T helper (Th) epitopes with CD8+ CTL epitopes theoretically generates a high efficacy vaccine as reviewed by Xu et al. [105]. HLA diversity data might be useful in predicting the relative population coverage of a specific vaccine, adding knowledge on epitope targets for vaccines [107], mechanisms of immune evasion [108, 109], and evaluation of drug efficacy [110]. Posteraro et al. reviewed the significance of HLA diversity in efficacy of vaccination, highlighting the need to further understand the link between genetic variation and immune responses [111].

It is generally easier to match donor-recipient pairs from populations with known HLA genotypes than in areas with information gaps [3], highlighting the need to understand population HLA diversity in order to improve donor-recipient matching. It is generally difficult to find a donor HLA match for patients of African descent owing to the paucity of Africans in global registries together with the occurrence of African specific alleles and or haplotypes and the high genetic diversity in these populations [60].

It is thus important to fully understand HLA diversity in the southern African context, to establish HLA-disease associations, to use this data for the informed design of population-specific vaccines against the many diseases, and to improve donor-recipient matching.

## Supplementary Material

A Microsoft Excel spreadsheet listing all classical HLA alleles, their frequencies as reported by the AFND and a limited number of disease association studies in southern African populations has been made available online as supplementary Material (S1)

## Figures and Tables

**Table:** (a) General description of studies used in this review

Country	Year	Population	*n*	Typing method	Loci typed	Comments
Bots	2005		55	SSP	DRB, DQB1	55 HIV negative compared to 74 HIV positive [7]

Moza	2010	Mostly Black	202	SSOP	A, B, DRB1	91.8% Black, rest admixture. Assane et al. [35, 48, 55]

RSA	2012	Black	200	SBT, SSP	A, B, C, DRB1	Blacks from different ethnolinguistic groups in RSA. Paximadis et al. [18, 35, 48]

RSA	2012	Caucasians	102	SBT, SSP	A, B, C, DRB1	English and Afrikaner ancestry. Paximadis et al. [18, 35, 48]

RSA	2002	Tamil/Natal	51	SSOP	A, B, C	Hammond [35, 48, 56]

RSA	2000	Black Zulu/Natal	100	SSOP	A, B	Could not distinguish A^*∗*^0301 from A^*∗*^0303N, and B^*∗*^0705 from B^*∗*^0706 [35, 37, 48, 57]

RSA	2006	Black/Tswana	41		A, B	Coetzee et al. [35, 48, 58]

RSA	2004	Black	112	SSP	DRB1, DQB1, DPB1	112 Sclerosis controls compared to cases [59]

Zam	2002	Black/Lusaka	44	SSOP	A, B, C	Alleles similar at exons 2 and 3 could not be distinguished [35, 48, 60, 61]

Zim	2002	Shona/Harare	230	SSOP	A, B, C, DPB1, DQA1, DQB1, DRB1	Louie [35, 48, 62]

**Table:** (b) Most frequent alleles in different southern African populations [35, 48]

Population	Loci					
	A	B	C	DP	DQ	DRB1
Black RSA	A^*∗*^30:01 (0.101)	B^*∗*^42:01 (0.089), B^*∗*^58:02 (0.094)	C^*∗*^06:02 (0.149)	DPB1^*∗*^13:01 (0.148) [59]	DQB1^^*∗*^^06 (0.555) [59]	DRB1^*∗*^11 (0.366) [59], DRB1^*∗*^13:01 (0.124)

Bots					DQB1^*∗*^16 (0.509) [7]	DRB1^*∗*^11 (0.364) [7]

Caucasian RSA	A^*∗*^01:01:01 (0.2), A^*∗*^02:01:01 (0.26)	B^*∗*^07:02:01 (0.149)	C^*∗*^07:01 (0.172), C^*∗*^07:02:01 (0.137)			DRB1^*∗*^03:01 (0.122)

Moza	A^*∗*^30 (0.239)	B^*∗*^15 (0.156)				DRB1^*∗*^11 (0.196), DRB1^*∗*^13 (0.198)

Shona Zim	A^*∗*^30:02 (0.147)	B^*∗*^45:01 and B^*∗*^53:01 (0.093)	C^*∗*^04:01 (0.148)	DPB1^*∗*^01:01:01 (0.355)	DQA1^*∗*^01:02 (0.343), DQB1^*∗*^05:01 (0.227), DQB1^*∗*^06:02 (0.247)	DRB1^*∗*^11:01 (0.144), DRB1^*∗*^15:03 (0.153)

Tamil RSA	A^*∗*^01:01 (0.17), A^*∗*^11:01 (0.18)	B^*∗*^40:06 (0.143)	C^*∗*^06:02 (0.177)			

Tswana RSA	A^*∗*^02 (0.146), A^*∗*^30 (0.159)	B^*∗*^58 (0.22)				

Venda RSA					DQB1^*∗*^06 (0.437)	DRB1^*∗*^11 (0.184)

Zam	A^*∗*^30:02 (0.233)	B^*∗*^42:01 (0.148)	C^*∗*^17:01 (0.156)			

Zulu RSA	A^*∗*^30 (0.195)	B^*∗*^15 (0.15), B^*∗*^58 (0.145)				

**Table:** (c) Least frequent alleles in different southern African populations [35, 48]

Population	Loci					
	A	B	C	DP	DQ	DRB1
Bots					DQB1^*∗*^02 (0.127) [7]	DRB1^*∗*^10 and DRB1^*∗*^12 (0.074) [7]

Caucasian RSA	A^*∗*^02:05, A^*∗*^02:17, A^*∗*^11:12, A^*∗*^24:07, A^*∗*^25:01:01, A^*∗*^33:03:01 and A^*∗*^69:01 (0.005)	B^*∗*^07:06, B^*∗*^14:01, B^*∗*^15:02, B^*∗*^15:03, B^*∗*^15:10, B^*∗*^15:13, B^*∗*^15:16, B^*∗*^15:24, B^*∗*^27:02, B^*∗*^35:05, B^*∗*^40:06:01, B^*∗*^41:01, B^*∗*^44:04, B^*∗*^44:27, B^*∗*^45:01, B^*∗*^49:01, B^*∗*^50:01 and B^*∗*^58:02 (0.005)	C^*∗*^02:05, C^*∗*^03:16, C^*∗*^04:08, C^*∗*^04:09N, C^*∗*^06:11, C^*∗*^07:22, C^*∗*^08:01, C^*∗*^14:04 and C^*∗*^17:01 (0.005)			DRB1^*∗*^03:02, DRB1^*∗*^04:08, DRB1^*∗*^12:02, DRB1^*∗*^14:04 and DRB1^*∗*^15:07 (0.005)

Moza	A^*∗*^32 (0.002)	B^*∗*^27, B^*∗*^37, B^*∗*^73 and B^*∗*^82 (0.002)				DRB1^*∗*^16 (0.002)

Shona Zim	A^*∗*^02:17, A^*∗*^32:02, A^*∗*^34:01, A^*∗*^80:01, A^*∗*^66:02, A^*∗*^66:03 and A^*∗*^74 (0.002)	B^*∗*^07:12, B^*∗*^13:04, B^*∗*^14:04, B^*∗*^15:17, B^*∗*^15:18, B^*∗*^35:02, B^*∗*^39:10, B^*∗*^40:01, B^*∗*^40:16, B^*∗*^50:02 and B^*∗*^73:01 (0.002)	C^*∗*^03:04:01, C^*∗*^07:08, C^*∗*^12:04:02 and C^*∗*^15:05 (0.02)	DPB1^*∗*^01:01:02, DPB1^*∗*^02:02, DPB1^*∗*^62:01, DPB1^*∗*^65:01 and DPB1^*∗*^80:01 (0.002)	DQA1^*∗*^05:02 (0.004), DQB1^*∗*^06:08 and DQB1^*∗*^06:15 (0.002)	DRB1^*∗*^03, DRB1^*∗*^04:04, DRB1^*∗*^12:04, DRB1^*∗*^13 and DRB1^*∗*^15:01 (0.002)

Tamil RSA	A^*∗*^02:01, A^*∗*^02:03, A^*∗*^03:02, A^*∗*^24:07, A^*∗*^30:01 and A^*∗*^32:01 (0.001)	B^*∗*^15:25, B^*∗*^27:05, B^*∗*^44:07, B^*∗*^50:01 and B^*∗*^56:01 (0.01)	C^*∗*^02:02:01, C^*∗*^12:03, C^*∗*^15:02 and C^*∗*^16:01 (0.01)			

Tswana RSA	A^*∗*^01, A^*∗*^31, A^*∗*^32, A^*∗*^36 and A^*∗*^80 (0.012)	B^*∗*^35, B^*∗*^40, B^*∗*^50 and B^*∗*^53 (0.012)				

Venda RSA					DQB1^*∗*^04 (0.094)	DRB1^*∗*^10:01 (0.004)

Zam	A^*∗*^02:06, A^*∗*^02:14, A^*∗*^26:01, A^*∗*^33:01, A^*∗*^34:02, A^*∗*^43:01 and A^*∗*^66:01 (0.012)	B^*∗*^07:05, B^*∗*^13:02, B^*∗*^15:18, B^*∗*^18:03, B^*∗*^41:01, B^*∗*^44:05, B^*∗*^47:01, B^*∗*^49:01 and B^*∗*^57:01 (0.011)	C^*∗*^03:03 and C^*∗*^07:04 (0.022)			

Zulu RSA	A^*∗*^31, A^*∗*^31:01:02, A^*∗*^33 and A^*∗*^33:03 (0.005)	B^*∗*^15:01, B^*∗*^15:16, B^*∗*^41:01, B^*∗*^41:02, B^*∗*^67, B^*∗*^67:01, B^*∗*^82 and B^*∗*^82:01 (0.005)				

*n* = sample size, Bots = Botswana, Moza = Mozambique, RSA = Republic of South Africa, Zam = Zambia, Zim = Zimbabwe, SSP = sequence specific primers, SBT = sequence based typing, SSOP = sequence specific oligonucleotide primers, and (number) is allele frequency in the population stated. Blanks indicate no alleles reported in the population or ethnicity not defined or typing method not specified.

**Table 2:** Number of classical HLA alleles reported in each geographical region. Sub-Saharan Africa (including southern Africa) generally has a high number of class I alleles (ranked in the top 5 regions) with a low number of class II alleles (ranked in the bottom 5 regions). More than half of the reported class I alleles in sub-Saharan Africa come from southern Africa, with less than half of the reported class II alleles in the sub-Saharan region coming from southern Africa, data from AFND [35, 48].

Region	HLA loci							
	A	B	C	DPA1	DPB1	DQA1	DPB1	DRB1
Australia	49	95	33	*∗*	20	12	17	40
Europe	714	1121	387	16	137	47	89	602
N. Africa	982	1559	600	*∗*	32	30	89	269
N. America	721	1166	390	7	74	29	93	574
N.E. Asia	262	477	131	12	78	47	57	318
S.Central America	121	288	59	12	78	28	60	549
S./S.E. Asia	407	731	227	10	99	21	64	280
Sub-Saharan Africa	154	313	94	12	87	23	48	220
W. Asia	215	366	167	*∗*	29	21	57	138
Oceania	163	256	85	16	84	10	48	93
Southern Africa	131	291	54	*∗*	21^a^	8^a^	20	58

N. Africa = North Africa, N. America = North America, N.E. Asia = North East Asia, S.Central America = South and Central America, S/S.E. Asia = South and South East Asia, and W. Asia = West Asia. ^*∗*^No loci specific alleles were reported in this region in the AFND. ^a^Alleles only reported in Zimbabwean black Shona population in the AFND.
