# Supplementary material for: Human Leukocyte Antigen Diversity: A Southern African Perspective
Source: J Immunol Res. 2015 Aug 12;2015:746151. doi: 10.1155/2015/746151 (PMC4549606; doi:10.1155/2015/746151)
Supplement: Supplementary file 1 — A Microsoft Excel spreadsheet listing all classical HLA alleles, their frequencies as reported by the AFND and a limited number of disease association studies in southern African populations has been made available online as supplementary Material (S1) [file 746151.f1.pdf]

HLA allele frequencies for southern Africa all were obtained from the allele frequency.net database (http://www.allelefrequencies.net/)

Frequency (number of individuals with allele)

allele	Mozambique n=202	Black RSA n=200	Caucasian RSA n=102	Natal Tamil RSA n=51	Natal Zulu RSA n=100	Tswana RSA n=41	Lusaka Zambia n=44	Shona Harare Zimbabwe n=230
A*01	0.024				0.04 (8)	0.012 (2.5)		
A*01:01				0.17	0.04 (8)		0.035	0.004
A*01:01:01		0.02	0.2					
A*01:03		0.003						
A*02	0.151				0.115 (23)	0.146 (24.4)		
A*02:01				0.01	0.025 (5)		0.128	0.091
A*02:01:01		0.083	0.26					
A*02:02		0.023	0.01		0.035 (7)		0.023	0.036
A*02:03				0.01				
A*02:05		0.068	0.005		0.055 (11)			0.031
A*02:06							0.012	
A*02:10				0.02				
A*02:11				0.13				
A*02:14		0.003					0.012	
A*02:17			0.005					0.002
A*03	0.057				0.06 (12)	0.085 (17.1)		
A*03:01				0.04	0.06 (12)		0.058	0.04
A*03:01:01		0.065	0.12					
A*03:02				0.01				
A*11	0.004							
A*11:01				0.18				
A*11:01:01		0.008	0.06					
A*11:12		0.003	0.005					
A*23	0.112				0.1 (20)	0.085 (14.6)		
A*23:01		0.08	0.015		0.1 (20)		0.081	0.127
A*24	0.018				0.01 (2)			
A*24:02								0.004
A*24:02:01		0.02	0.07					
A*24:02:01:01				0.16	0.01 (2)			
A*24:07			0.005	0.01				
A*25:01:01			0.005					
A*26	0.004				0.01 (2)	0.024 (4.9)		
A*26:01		0.008	0.025	0.02	0.01 (2)		0.012	0.016
A*29	0.099				0.125 (24)	0.122 (24.4)		
A*29:01					0.015 (3)			0.004
A*29:01:01		0.005						

A*29:02					0.11 (22)		0.058	0.096
A*29:02:01		0.063	0.06					
A*29:11		0.025						
A*30	0.239				0.195 (36)	0.159 (31.7)		
A*30:01		0.101	0.015	0.01	0.095 (18)		0.14	0.091
A*30:01:02		0.005						
A*30:02		0.096	0.005		0.085 (17)		0.233	0.147
A*30:02:02		0.005						
A*30:04		0.018	0.025		0.015 (3)			0.011
A*31					0.005 (1)	0.012 (2.4)		
A*31:01:02		0.003	0.015	0.07	0.005 (1)		0.035	
A*31:03								0.007
A*32	0.002				0.025 (5)	0.012 (2.4)		
A*32:01		0.013	0.03	0.01	0.025 (5)			0.009
A*32:02								0.002
A*32:03								0.004
A*33	0.044				0.005 (1)			
A*33:01		0.003	0.01				0.012	
A*33:03				0.07	0.005 (1)			0.024
A*33:03:01		0.018	0.005					
A*34	0.024				0.055 (11)	0.073 (14.6)		
A*34:01								0.002
A*34:02		0.035	0.02		0.055 (11)		0.012	0.033
A*36	0.029					0.012 (2.4)		
A*36:01		0.01					0.035	0.029
A*43	0.002				0.03 (6)	0.049 (9.7)		
A*43:01		0.035			0.03 (6)		0.012	0.004
A*66	0.039				0.035 (7)	0.037 (7.3)		
A*66:01		0.008			0.02 (4)		0.012	0.038
A*66:02		0.005			0.015 (3)			0.002
A*66:03								0.002
A*68	0.101				0.125 (23)	0.122 (24.4)		
A*68:01								0.009
A*68:01:01		0.033	0.015		0.03 (6)			0.004
A*68:01:02				0.08				
A*68:02		0.085	0.01		0.095 (19)		0.047	0.098
A*68:27		0.005						
A*69:01			0.005					
A*74	0.044				0.065 (13)	0.037 (4.9)		0.002
A*74:01		0.045					0.023	0.027

A*80	0.007					0.012 (2.4)		
A*80:01		0.005					0.023	0.002
B*07	0.029				0.065 (13)	0.024 (4.9)		
B*07:02					0.045 (9)		0.046	0.046
B*07:02:01		0.046	0.149					
B*07:05				0.041	0.02 (4)		0.011	
B*07:06		0.015	0.005					
B*07:12								0.002
B*08	0.077				0.075 (14)	0.037 (4.9)		
B*08:01		0.064	0.133	0.071	0.075 (14)		0.034	0.031
B*08:05								0.013
B*13	0.011				0.015 (3)	0.061 (12.2)		
B*13:01				0.061				
B*13:02		0.02	0.01		0.015 (3)		0.011	0.022
B*13:04								0.002
B*14	0.038				0.065 (13)	0.061 (12.2)		
B*14:01		0.018	0.005		0.04 (8)		0.046	0.013
B*14:02		0.015	0.021		0.025 (5)		0.034	0.024
B*14:04								0.002
B*15	0.156				0.15 (30)	0.159 (26.8)		
B*15:01					0.005 (1)		0.023	
B*15:01:01		0.005	0.087					
B*15:02			0.005	0.031				
B*15:03		0.084	0.005		0.095 (18)		0.057	0.077
B*15:05				0.02				
B*15:10		0.079	0.005		0.085 (16)		0.046	0.084
B*15:13			0.005					
B*15:16		0.02	0.005		0.005 (1)			0.013
B*15:17				0.031				0.002
B*15:17:01			0.01					
B*15:18				0.041			0.011	0.002
B*15:24			0.005					
B*15:25				0.01				
B*15:37								0.004
B*18	0.052				0.02 (4)	0.049 (7.3)		
B*18:01				0.031	0.02 (4)		0.034	0.064
B*18:01:01		0.031	0.051					
B*18:03							0.011	
B*27	0.002							
B*27:02			0.005					
B*27:05			0.041	0.01				

B*35	0.05				0.04 (8)	0.012 (2.4)		
B*35:01				0.02	0.04 (8)		0.023	0.013
B*35:01:01		0.018	0.062					
B*35:02								0.002
B*35:03				0.071				
B*35:05			0.005					
B*35:08			0.01					
B*35:20								0.004
B*37	0.002							
B*37:01			0.01					
B*37:01:01				0.02				
B*38:01			0.015					
B*39	0.018				0.015 (3)			
B*39:10		0.025			0.015 (3)			0.002
B*40	0.005					0.012 (2.4)		
B*40:01		0.003	0.021				0.023	0.002
B*40:02			0.01					
B*40:06				0.143				
B*40:06:01			0.005					
B*40:16								0.002
B*41	0.009				0.01 (2)			
B*41:01		0.015	0.005		0.005 (1)		0.011	0.004
B*41:02					0.005 (1)		0.023	
B*42	0.093				0.12 (23)	0.085 (17.1)		
B*42:01		0.089			0.12 (23)		0.148	0.069
B*42:02		0.015						0.011
B*42:06		0.003						
B*44	0.079				0.105 (20)	0.111 (22)		
B*44:02:01			0.056					
B*44:03				0.051	0.105 (20)		0.023	0.058
B*44:03:01		0.025	0.056					0.013
B*44:03:02		0.033	0.015					
B*44:04			0.005					
B*44:05			0.01				0.011	
B*44:07				0.01				
B*44:27			0.005					
B*45	0.061				0.035 (7)	0.11 (19.5)		
B*45:01		0.064	0.005		0.035 (7)		0.046	0.093
B*45:07		0.005						
B*47:01							0.011	
B*49	0.005				0.01 (2)			
B*49:01		0.008	0.005		0.01 (2)		0.011	0.009
B*50	0.005					0.012 (2.4)		

B*50:01			0.005	0.01				0.007
B*50:02								0.002
B*51	0.005							
B*51:01							0.057	0.004
B*51:01:01		0.01	0.046	0.092				
B*51:06				0.02				
B*52	0.005							
B*52:01								
B*52:01:01			0.01	0.082				
B*53	0.081				0.015 (3)	0.012 (2.4)		
B*53:01		0.043			0.015 (3)		0.102	0.093
B*55:01			0.015					
B*56:01				0.01				
B*57	0.05				0.05 (9)			
B*57:01				0.102			0.011	0.004
B*57:01:01			0.041					
B*57:02		0.015			0.01 (2)		0.023	0.007
B*57:03					0.04 (4)		0.057	0.044
B*57:03:01		0.023						
B*58	0.133				0.145 (28)	0.22 (39)		
B*58:01		0.081	0.026	0.02	0.04 (8)			0.044
B*58:02		0.094	0.005		0.085 (17)		0.023	0.064
B*67					0.005 (1)			
B*67:01					0.005 (1)			
B*73	0.002							
B*73:01								0.002
B*81	0.029				0.035 (7)	0.037 (7.3)		
B*81:01		0.033			0.035 (7)		0.023	0.04
B*82	0.002				0.005 (1)			
B*82:01					0.005 (1)			
C*01:02			0.02	0.021				
C*02:02		0.091	0.069				0.1	0.082
C*02:02:01				0.01				
C*02:05		0.003	0.005					
C*02:10								0.029
C*03:02		0.015		0.021				0.011
C*03:03		0.003	0.059	0.042			0.022	0.009
C*03:04		0.051	0.054				0.067	0.064
C*03:04:01								0.002
C*03:04:02								0.018
C*03:16			0.005					
C*04:01		0.119	0.088	0.042			0.144	0.148
C*04:03				0.031				
C*04:04		0.005						
C*04:07				0.042				0.015

C*04:08			0.005					
C*04:09N			0.005					
C*05:01								0.007
C*05:01:01		0.008	0.054					
C*06:02		0.149	0.078	0.177			0.033	0.128
C*06:06		0.003						
C*06:11			0.005					
C*07:01		0.076	0.172	0.135			0.122	0.106
C*07:02				0.094			0.044	0.046
C*07:02:01		0.058	0.137					
C*07:04		0.013	0.01	0.031			0.022	0.042
C*07:06		0.041	0.02					
C*07:08								0.002
C*07:11		0.003						
C*07:12		0.003						
C*07:18		0.041	0.015					
C*07:22			0.005					
C*08:01		0.003	0.005	0.042				0.029
C*08:02		0.023	0.025				0.067	0.038
C*08:04		0.025	0.01					
C*12:02			0.01	0.083				
C*12:02:03				0.021				
C*12:03		0.015	0.025	0.01				
C*12:04:02								0.002
C*14								
C*14:02		0.01	0.01	0.021				0.004
C*14:03				0.021				0.004
C*14:04			0.005					
C*15:02		0.008	0.029	0.104				
C*15:05		0.013						0.002
C*15:07				0.01				
C*16:01		0.066	0.064	0.01			0.1	0.08
C*16:02			0.01	0.031				
C*17:01		0.111	0.005				0.156	0.082
C*18:01		0.015					0.1	0.049
C*18:02		0.033						

HLA allele frequencies for southern Africa (*) were obtained from the allele frequency.net database (http://www.allelefrequencies.net/)
(#) were obtained from disease association studies (refs 6 and 66)

Frequency

Allele	Mozambique n=202*	Black RSA n=200*	Caucasian RSA n=102*	Limpopo Venda RSA n=117*	Shona Harare Zimbabwe n=230*	Botswana n=55#	RSA Tikley n=10#	
DRB1*01	0.051			0.103		16 (0.291)	9 (0.08)	
DRB1*01:01		0.013	0.075		0.02			
DRB1*01:02		0.047	0.011		0.061			
DRB1*01:03			0.021					
DRB1*03	0.181				0.002	12 (0.218)	35 (0.313)	
DRB1*03:01		0.076	0.122	0.137	0.07			
DRB1*03:02		0.097	0.005	0.09	0.066			
DRB1*03:04				0.077				
DRB1*03:05					0.009			
DRB1*03:07		0.003						
DRB1*03:18		0.005						
DRB1*04	0.021			0.064		7 (0.127)	9 (0.08)	
DRB1*04:01		0.026	0.112					
DRB1*04:02		0.003						
DRB1*04:03:01			0.016					
DRB1*04:04		0.018	0.053		0.002			
DRB1*04:05		0.005	0.011		0.022			
DRB1*04:07		0.003	0.027					
DRB1*04:08			0.005					
DRB1*04:10		0.003						
DRB1*07	0.06			0.056		5 (0.091)	14 (0.125)	
DRB1*07:01		0.071	0.096		0.046			
DRB1*08	0.045					8 (0.145)	10 (0.089)	
DRB1*08:01		0.005	0.011					
DRB1*08:02		0.003						
DRB1*08:04		0.042	0.011		0.046			
DRB1*09	0.009					0 (0.0)	2 (0.018)	
DRB1*09:01				0.009	0.015			
DRB1*09:01:02		0.005	0.021					
DRB1*10	0.06					4 (0.073)	2 (0.018)	
DRB1*10:01		0.026		0.004	0.037			
DRB1*11	0.196			0.184		20 (0.364)	41 (0.366)	
DRB1*11:01		0.116	0.059		0.144			
DRB1*11:02		0.079			0.046			
DRB1*11:04		0.003	0.037		0.007			
DRB1*11:16		0.003						
DRB1*11:19		0.003						
DRB1*12	0.04			0.013		4 (0.073)	11 (0.098)	
DRB1*12:01		0.026	0.011		0.052			

DRB1*12:02			0.005		0.007			
DRB1*12:03:02					0.004			
DRB1*12:04					0.002			
DRB1*13	0.198				0.002	16 (0.291)	39 (0.348)	
DRB1*13:01		0.124	0.064	0.128	0.085			
DRB1*13:02		0.058	0.037	0.03	0.066			
DRB1*13:03		0.018	0.016		0.009			
DRB1*14	0.013						1 (0.009)	
DRB1*14:01		0.008	0.027		0.015			
DRB1*14:04			0.005					
DRB1*14:15					0.002			
DRB1*15	0.126			0.073				
DRB1*15:01		0.024	0.112		0.002			
DRB1*15:03		0.084	0.011		0.153			
DRB1*15:07			0.005					
DRB1*16	0.002			0.034				
DRB1*16:01			0.016					
DRB1*16:02		0.003			0.009			
DRB3*01						19 (0.345)		
DRB3*02						18 (0.327)		
DRB3*03						16 (0.291)		
DRB4*01						9 (0.164)		
DRB5*01						12 (0.218)		
DQB1*02				0.111		7 (0.127)	26 (0.262)	
DQB1*02:01					0.133			
DQB1*03				0.124		22 (0.40)		
DQB1*03:01					0.14			
DQB1*03:02					0.022			
DQB1*03:03					0.009			
DQB1*04				0.094		13 (0.236)	19 (0.192)	
DQB1*04:02					0.094			
DQB1*05				0.235		13 (0.236)	30 (0.303)	
DQB1*05:01					0.227			
DQB1*05:02					0.011			
DQB1*05:03					0.007			
DQB1*06				0.437		28 (0.509)	55 (0.555)	
DQB1*06:02					0.247		27 (0.31)	
DQB1*06:03					0.052		8 (0.092)	
DQB1*06:04					0.024			
DQB1*06:08					0.002			
DQB1*06:09					0.022			
DQB1*06:13					0.009			
DQB1*06:15					0.002			
DQB1*07							29 (0.293)	
DQB1*08							5 (0.05)	
DQA1*01:01					0.197			
DQA1*01:02					0.343			

DQA1*01:03					0.066				
DQA1*02:01					0.037				
DQA1*03:01:01					0.059				
DQA1*04:01					0.111				
DQA1*05:01					0.183				
DQA1*05:02					0.004				
DPB1*01:01:01					0.355				
DPB1*01:01:02					0.002				
DPB1*02:01:02					0.107				
DPB1*02:02					0.002				
DPB1*03:01					0.064				
DPB1*04:01					0.031				
DPB1*04:02					0.178				
DPB1*11:01					0.018				
DPB1*13:01					0.044		9 (0.148)		
DPB1*14:01					0.011				
DPB1*15:01					0.007				
DPB1*17:01					0.031				
DPB1*18:01					0.099				
DPB1*19:01					0.013				
DPB1*34:01					0.004				
DPB1*39:01					0.013				
DPB1*55:01					0.011				
DPB1*62:01					0.002				
DPB1*65:01					0.002				
DPB1*75:01					0.004				
DPB1*80:01					0.002				
